# Supplementary material for: Thiabendazole and Thiabendazole-Formic Acid Solvate: A Computational, Crystallographic, Spectroscopic and Thermal Study
Source: Molecules. 2020 Jul 6;25(13):3083. doi: 10.3390/molecules25133083 (PMC7412198; doi:10.3390/molecules25133083)
Supplement: Supplementary file 1 [file molecules-25-03083-s001.pdf]

Thiabendazole and Thiobendazole-Formic Acid Solvate: A Computational, Crystallographic, Spectroscopic and Thermal Study

Andreia M. Tabanez,[1] Bernardo A. Nogueira,[1,2,*] Alberto Milani,[2] M. Ermelinda Eusébio,[1] José A. Paixão,[3] Hayrunnisa Nur Kabuk,[4] Maria Jajuga,[1] Gulce O. Ildiz[1,4] and Rui Fausto[1,5]

[1] *University of Coimbra, CQC, Department of Chemistry, P-3004-535 Coimbra, Portugal.*

[2] *CMIC, Dipartimento di Chimica, Materiali e Ingegneria Chimica "G. Natta", Politecnico di Milano, Italy.*

[3] *University of Coimbra, CFisUC, Department of Physics, P-3004-516 Coimbra, Portugal.*

[4] *Department of Physics, Faculty of Sciences and Letters, Istanbul Kultur University, Turkey.*

[5] *Department of Chemistry, King Fahd University of Petroleum and Minerals, 31261 Dhahran, Saudi Arabia.*

Supporting Information

Contents

Figure S1 - TG and Powder XRD Results for TBZ 2
Figure S2 - Periodic B3LYP-D2/6-31G(d,p) calculated infrared spectrum of TBZ-formic acid solvate crystal 3
Table S1 - Crystal Data and Details of the Structure Determination for TBZ-Formic Acid Solvate 4
Table S2 - Final Coordinates and Equivalent Isotropic Displacement Parameters of the non-Hydrogen Atoms 5
Table S3 - Hydrogen Atom Positions and Isotropic Displacement Parameters 6
Table S4 - (An)isotropic Displacement Parameters 7
Table S5 - Bond Distances 8
Table S6 - Bond Angles 9
Table S7 - Torsion Angles 10
Table S8 - Contact Distances 11
Table S9 - Hydrogen Bonds 13

* Corresponding author e-mail: ban@qui.uc.pt

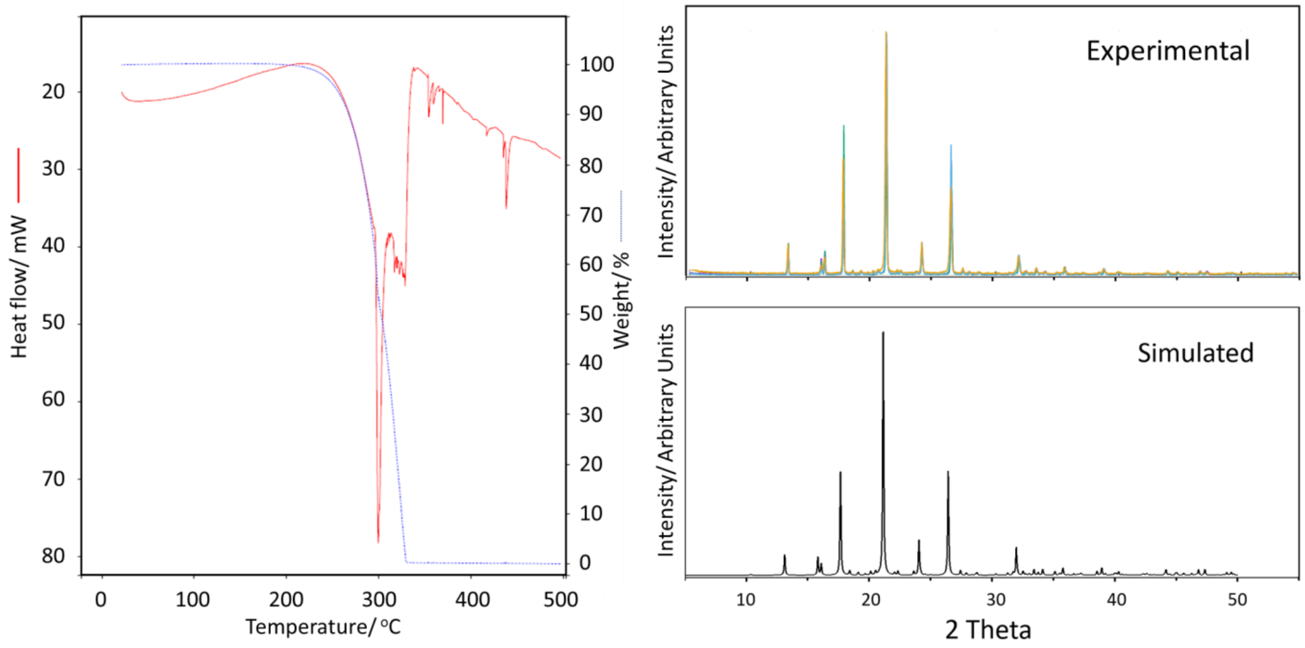

Figure S1 – *Left*: TG results on TBZ (12.987 mg; heat from 20.00 °C to 500.00 °C at 10.00 °C/min). *Right-top*: powder XRD experimental data for TBZ data collected for the virgin sample, for two samples heated up to 290 °C and 310 °C, respectively, and for the sublimated material collected on the top plate of the DSC/TG furnace; the data are virtually equal. *Right-bottom*: simulated powder XRD data for TBZ using the structural data reported in Ref 11.

Powder X-ray Diffraction

The powder x-ray diffraction (XRD) patterns (Bragg-Brentano geometry) were collected on a Bruker D8 Advance diffractometer equipped with a silicon drift 1D LYNXEYE detector using Cu Kda radiation (λ =1.54056 Å). A K_β filter (Ni foil) was used before the detector, and 2.5° Soller slits at the incident and diffracted beams. Data were collected with a 0.2° divergence slit, a 0.01° angular step and 0.5 s/point counting time. The sample holder was a "zero-background" off-cut Si single crystal and a knife-edge was placed close to the flat sample to further reduce the background. XRD data were collected for the virgin sample, for two samples heated up to 290 °C and 310 °C and for the sublimated material collected on the top plate of the differential scanning calorimetry/thermal gravimetry (DSC/TG) furnace.

STA (DSC/TG)

The simultaneous thermal analysis (STA: DSC/TG) data were measured on a Perkin-Elmer STA6000 instrument using a heating rate of 10 K/min, with the sample kept under a 20 ml/min N_2 flow atmosphere. Around 15 mg of compound for each sample were placed in alumina crucibles. Calibration of enthalpies was performed using pure In and Ag standards. Thermogravimetric curves were measured up to 290 °C, 300 °C, 310 °C and 500 °C, always on fresh samples, with identical results.

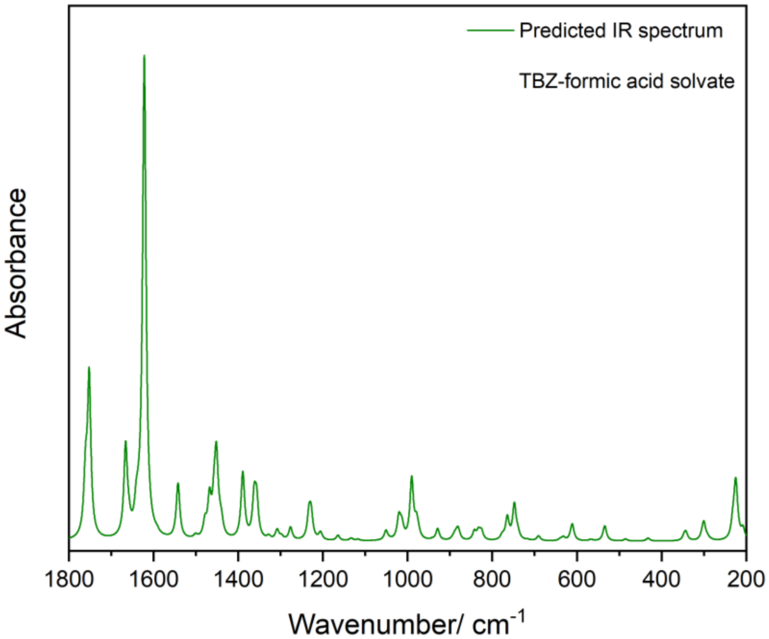

Figure S2. Periodic B3LYP-D2/6-31G(d,p) calculated infrared spectrum of TBZ-formic acid solvate crystal. Predicted frequencies are scaled by 0.972.

Table S1 - Crystal Data and Details of the Structure Determination

Crystal Data for TBZ- Formic Acid Solvate

Formula	C10 H8 N3 S +, C H O2 -, C H2 O2		
Formula Weight			293.30
Crystal System			monoclinic
Space group		P21/c	(No. 14)
a, b, c [Angstrom]	3.8339(1)	22.1950(6)	15.3695(4)
alpha, beta, gamma [deg]	90	93.412(1)	90
V [Ang**3]			1305.53(6)
Z			4
D(calc) [g/cm**3]			1.492
Mu(MoKa) [/mm]			0.265
F(000)			608
Crystal Size [mm]			0.22 x 0.27 x 0.35
Data Collection			
Temperature (K)			293
Radiation [Angstrom]		MoKa	0.71073
Theta Min-Max [Deg]			2.7, 28.3
Dataset			-5: 5 ; -29: 29 ; -20: 20
Tot., Uniq. Data, R(int)			37466, 3234, 0.053
Observed Data [I > 2.0 sigma(I)]			2190
Refinement			
Nref, Npar			3234, 190
R, wR2, S			0.0477, 0.1262, 1.02
w = ^2^(FO^2^)+(0.0509P)^2^+0.6789P] WHERE P=(FO^2^+2FC^2^)/3'			
Max. and Av. Shift/Error			0.00, 0.00
Min. and Max. Resd. Dens. [e/Ang^3]			-0.25, 0.33

Table S2 - Final Coordinates and Equivalent Isotropic Displacement Parameters of the non-Hydrogen atoms

Crystal Data for TBZ- Formic Acid Solvate

Atom	x	y	z	U(eq) [Ang^2]
S	0.21977(18)	0.42480(3)	0.53049(4)	0.0515(2)
N1	0.4171(5)	0.23873(8)	0.40285(11)	0.0361(5)
N2	0.1253(5)	0.32000(8)	0.59360(12)	0.0448(6)
N3	0.2037(5)	0.20232(8)	0.51969(11)	0.0359(5)
C1	0.1033(6)	0.37710(10)	0.61016(15)	0.0471(8)
O1	0.9074(5)	0.32947(7)	0.16673(10)	0.0514(6)
C2	0.3110(6)	0.36466(10)	0.46828(15)	0.0433(7)
O2	0.7022(5)	0.31338(7)	0.29570(10)	0.0532(6)
C3	0.2461(5)	0.31279(9)	0.51119(13)	0.0351(6)
C4	0.2885(5)	0.25233(9)	0.47856(12)	0.0337(6)
C5	0.2816(5)	0.15321(9)	0.46826(13)	0.0352(6)
C6	0.4148(5)	0.17639(9)	0.39372(13)	0.0351(6)
C7	0.5140(6)	0.13935(11)	0.32747(14)	0.0437(7)
C8	0.4747(6)	0.07880(11)	0.33881(16)	0.0491(8)
C9	0.3447(6)	0.05416(11)	0.41440(15)	0.0468(8)
C10	0.2458(6)	0.09200(10)	0.48076(15)	0.0441(7)
O3	1.1154(6)	0.49910(9)	0.32259(14)	0.0757(8)
O4	1.1456(7)	0.43313(9)	0.21881(16)	0.0851(9)
C11	0.7619(6)	0.29835(11)	0.22140(14)	0.0460(8)
C12	1.0161(7)	0.45477(11)	0.28566(16)	0.0520(8)

U(eq) = 1/3 of the trace of the orthogonalized U Tensor

Table S3 - Hydrogen Atom Positions and Isotropic Displacement Parameters

Crystal Data for TBZ- Formic Acid Solvate

Atom	x	y	z	U(iso) [Ang^2]
H1	0.495(6)	0.2663(11)	0.3689(15)	0.0430
H1A	0.02784	0.39119	0.66284	0.0570
H2	0.39362	0.36682	0.41269	0.0520
H3	0.105(6)	0.2009(10)	0.5658(16)	0.0430
H7	0.60380	0.15496	0.27729	0.0520
H8	0.53645	0.05289	0.29473	0.0590
H9	0.32457	0.01261	0.42015	0.0560
H10	0.15918	0.07667	0.53149	0.0530
H11	0.69151	0.25986	0.20407	0.0550
H4	1.060(11)	0.397(2)	0.202(3)	0.1280
H12	0.82856	0.43441	0.30768	0.0620

The Temperature Factor has the Form of Exp(-T) Where

T = 8*(Pi**2)*U*(Sin(Theta)/Lambda)**2 for Isotropic Atoms

Table S4 - (An)isotropic Displacement Parameters

Crystal Data for TBZ- Formic Acid Solvate

Atom	U(1,1) or U	U(2,2)	U(3,3)	U(2,3)	U(1,3)	U(1,2)
----	------	------	------	------	------	------
S	0.0640(4)	0.0368(3)	0.0549(4)	-0.0011(3)	0.0141(3)	0.0016(3)
N1	0.0422(10)	0.0402(10)	0.0269(8)	0.0049(7)	0.0118(7)	0.0006(8)
N2	0.0607(12)	0.0423(10)	0.0323(9)	0.0011(8)	0.0116(9)	0.0062(9)
N3	0.0446(10)	0.0399(9)	0.0243(8)	0.0035(7)	0.0113(7)	0.0018(8)
C1	0.0617(15)	0.0456(13)	0.0350(12)	-0.0069(10)	0.0105(11)	0.0061(11)
O1	0.0768(12)	0.0476(9)	0.0326(8)	-0.0037(7)	0.0270(8)	-0.0085(8)
C2	0.0509(13)	0.0434(12)	0.0369(11)	0.0015(9)	0.0141(10)	0.0044(10)
O2	0.0759(12)	0.0536(10)	0.0329(8)	0.0025(7)	0.0256(8)	-0.0094(8)
C3	0.0374(11)	0.0383(11)	0.0298(10)	-0.0012(8)	0.0039(8)	0.0044(9)
C4	0.0348(10)	0.0412(11)	0.0255(9)	0.0032(8)	0.0052(8)	0.0033(9)
C5	0.0353(11)	0.0399(11)	0.0306(10)	-0.0007(8)	0.0042(8)	0.0029(9)
C6	0.0356(11)	0.0387(11)	0.0312(10)	-0.0011(8)	0.0050(8)	0.0035(9)
C7	0.0469(13)	0.0522(14)	0.0327(11)	-0.0059(9)	0.0087(10)	0.0040(10)
C8	0.0513(14)	0.0511(14)	0.0450(13)	-0.0165(11)	0.0031(11)	0.0092(11)
C9	0.0552(14)	0.0393(12)	0.0456(13)	-0.0044(10)	0.0018(11)	0.0014(10)
C10	0.0528(14)	0.0399(12)	0.0403(12)	0.0039(9)	0.0086(10)	0.0006(10)
O3	0.1091(17)	0.0525(12)	0.0671(13)	-0.0164(10)	0.0195(12)	-0.0130(11)
O4	0.1196(19)	0.0541(12)	0.0877(16)	-0.0214(11)	0.0586(14)	-0.0284(12)
C11	0.0616(15)	0.0422(12)	0.0356(12)	-0.0023(9)	0.0157(11)	-0.0080(11)
C12	0.0612(16)	0.0476(14)	0.0482(14)	0.0013(12)	0.0127(12)	-0.0024(12)

===

The Temperature Factor has the Form of Exp(-T) Where

T = 8*(Pi**2)*U*(Sin(Theta)/Lambda)**2 for Isotropic Atoms

T = 2*(Pi**2)*Sumij(h(i)*h(j)*U(i,j)*Astar(i)*Astar(j)), for Anisotropic Atoms. Astar(i) are Reciprocal Axial Lengths and h(i) are the Reflection Indices.

Table S5 - Bond Distances (Angstrom)

Crystal Data for TBZ- Formic Acid Solvate

S	-C1	1.698(2)	C6	-C7	1.380(3)
S	-C2	1.691(2)	C7	-C8	1.365(3)
N1	-C4	1.325(3)	C8	-C9	1.402(3)
N1	-C6	1.391(3)	C9	-C10	1.391(3)
N2	-C1	1.296(3)	C1	-H1A	0.9300
N2	-C3	1.384(3)	C2	-H2	0.9300
N3	-C4	1.327(3)	C7	-H7	0.9300
N3	-C5	1.389(3)	C8	-H8	0.9300
O1	-C11	1.245(3)	C9	-H9	0.9300
N1	-H1	0.87(2)	C10	-H10	0.9300
C2	-C3	1.357(3)	O3	-C12	1.187(3)
O2	-C11	1.224(3)	O4	-C12	1.262(3)
N3	-H3	0.82(2)	O4	-H4	0.90(4)
C3	-C4	1.445(3)	C11	-H11	0.9300
C5	-C10	1.380(3)	C12	-H12	0.9300
C5	-C6	1.381(3)			

Table S6 - Bond Angles (Degrees)

Crystal Data for TBZ- Formic Acid Solvate

C1	-S	-C2	89.30(11)	C7	-C8	-C9	122.5(2)
C4	-N1	-C6	108.35(17)	C8	-C9	-C10	119.9(2)
C1	-N2	-C3	108.79(18)	C5	-C10	-C9	117.3(2)
C4	-N3	-C5	108.55(17)	S	-C1	-H1A	122.00
S	-C1	-N2	116.41(17)	N2	-C1	-H1A	122.00
C6	-N1	-H1	129.8(16)	S	-C2	-H2	125.00
C4	-N1	-H1	121.8(16)	C3	-C2	-H2	125.00
S	-C2	-C3	110.17(17)	C6	-C7	-H7	121.00
N2	-C3	-C2	115.33(19)	C8	-C7	-H7	121.00
C4	-N3	-H3	125.4(16)	C7	-C8	-H8	119.00
C5	-N3	-H3	125.8(16)	C9	-C8	-H8	119.00
N2	-C3	-C4	118.41(17)	C8	-C9	-H9	120.00
C2	-C3	-C4	126.26(19)	C10	-C9	-H9	120.00
N3	-C4	-C3	125.13(17)	C5	-C10	-H10	121.00
N1	-C4	-N3	110.01(18)	C9	-C10	-H10	121.00
N1	-C4	-C3	124.86(18)	O1	-C11	-O2	126.5(2)
N3	-C5	-C10	131.81(19)	C12	-O4	-H4	115(3)
C6	-C5	-C10	121.80(19)	O2	-C11	-H11	117.00
N3	-C5	-C6	106.40(17)	O1	-C11	-H11	117.00
N1	-C6	-C5	106.70(17)	O3	-C12	-O4	125.0(3)
N1	-C6	-C7	131.83(19)	O3	-C12	-H12	117.00
C5	-C6	-C7	121.47(19)	O4	-C12	-H12	117.00
C6	-C7	-C8	117.1(2)				

Table S7 - Torsion Angles (Degrees)

Crystal Data for TBZ- Formic Acid Solvate

C2	-S	-C1	-N2	-0.4(2)
C1	-S	-C2	-C3	0.36(18)
C6	-N1	-C4	-N3	0.3(2)
C6	-N1	-C4	-C3	-179.50(18)
C4	-N1	-C6	-C5	-0.6(2)
C4	-N1	-C6	-C7	178.9(2)
C3	-N2	-C1	-S	0.3(2)
C1	-N2	-C3	-C2	-0.1(3)
C1	-N2	-C3	-C4	-179.51(19)
C5	-N3	-C4	-N1	0.1(2)
C5	-N3	-C4	-C3	179.94(18)
C4	-N3	-C5	-C6	-0.5(2)
C4	-N3	-C5	-C10	179.7(2)
S	-C2	-C3	-N2	-0.3(2)
S	-C2	-C3	-C4	179.16(17)
N2	-C3	-C4	-N1	-176.60(19)
N2	-C3	-C4	-N3	3.6(3)
C2	-C3	-C4	-N1	4.0(3)
C2	-C3	-C4	-N3	-175.8(2)
N3	-C5	-C6	-N1	0.7(2)
N3	-C5	-C6	-C7	-178.86(19)
C10	-C5	-C6	-N1	-179.51(19)
C10	-C5	-C6	-C7	1.0(3)
N3	-C5	-C10	-C9	178.8(2)
C6	-C5	-C10	-C9	-1.0(3)
N1	-C6	-C7	-C8	-179.4(2)
C5	-C6	-C7	-C8	0.0(3)
C6	-C7	-C8	-C9	-0.9(3)
C7	-C8	-C9	-C10	0.9(4)
C8	-C9	-C10	-C5	0.1(3)

Table S8 - Contact Distances (Angstrom)

Crystal Data for TBZ- Formic Acid Solvate

S	.C1_a	3.688(2)	O3	.H8_k	2.6000
S	.C3	2.506(2)	N3	.C6	2.218(3)
S	.O3_b	3.154(2)	O3	.H1A_l	2.5100
O1	.C11_h	3.404(3)	O4	.H12_m	2.8800
O1	.C12	3.340(3)	O4	.H10_i	2.8900
O1	.N3_i	2.684(2)	N1	.H7	2.8000
O1	.O4	2.585(3)	N1	.H2	2.8500
O2	.N1	2.620(2)	C1	.O3_b	3.070(3)
O2	.C12	3.368(3)	C1	.S_d	3.688(2)
O2	.C2	3.325(3)	C1	.C3	2.180(3)
O3	.S_l	3.154(2)	C1	.C4	3.526(3)
O3	.C1_l	3.070(3)	C2	.C12_e	3.575(3)
O4	.C11	3.335(3)	N2	.H3	2.68(2)
O4	.O1	2.585(3)	C2	.O2	3.325(3)
O1	.H4	1.69(4)	C2	.N2	2.316(3)
O1	.H3_i	1.89(2)	C2	.N1	3.006(3)
N1	.C11	3.423(3)	N3	.H10	2.8000
N1	.C5	2.224(3)	C3	.S	2.506(2)
N1	.C2	3.006(3)	C4	.C5	2.206(3)
N1	.O2	2.620(2)	C4	.C1	3.526(3)
O2	.H2	2.5100	C4	.C10	3.563(3)
O2	.H4	2.76(4)	C4	.C7	3.559(3)
O2	.H1	1.76(2)	C5	.C6_d	3.488(3)
N2	.C2	2.316(3)	C5	.C7_d	3.561(3)
O2	.H12	2.7300	C5	.C8	2.722(3)
N2	.N3	2.871(3)	C5	.N1	2.224(3)
N3	.O1_c	2.684(2)	C6	.N3	2.218(3)
N3	.N2	2.871(3)	C6	.C9	2.747(3)
C6	.C5_a	3.488(3)	C11	.H3_i	2.80(2)
C7	.C4	3.559(3)	C11	.H1	2.64(2)
C7	.C5_a	3.561(3)	C12	.H2_a	3.0600
C7	.C10	2.829(3)	C12	.H8_k	3.0800

Table S8 - Contact Distances(Angstrom) (continued)

Crystal Data for TBZ- Formic Acid Solvate

C8	.C9_a	3.509(3)	H1	.C2	2.78(2)
C8	.C10_a	3.580(3)	H1	.C3	2.65(2)
C8	.C5	2.722(3)	H1	.C5	3.07(2)
C9	.C8_d	3.509(3)	H1	.C7	2.89(2)
C9	.C6	2.747(3)	H1	.C11	2.64(2)
C10	.C8_d	3.580(3)	H1	.H2	2.3700
C10	.C4	3.563(3)	H1	.O2	1.76(2)
C10	.C7	2.829(3)	H1A	.C3	3.0600
C11	.O1_j	3.404(3)	H1A	.O3_b	2.5100
C11	.N1	3.423(3)	H2	.N1	2.8500
C11	.O4	3.335(3)	H2	.C4	2.7700
C12	.C2_a	3.575(3)	H2	.C12_e	3.0600
C12	.O2	3.368(3)	H2	.H1	2.3700
C12	.O1	3.340(3)	H2	.O2	2.5100
C2	.H1	2.78(2)	H3	.N2	2.68(2)
C3	.H1A	3.0600	H3	.C3	2.69(2)
C3	.H1	2.65(2)	H3	.C10	2.82(2)
C3	.H3	2.69(2)	H3	.O1_c	1.89(2)
C4	.H2	2.7700	H3	.C11_c	2.80(2)
C5	.H1	3.07(2)	H3	.C6	3.01(2)
C6	.H3	3.01(2)	H4	.H12	2.0700
C7	.H1	2.89(2)	H4	.O1	1.69(4)
C10	.H3	2.82(2)	H4	.O2	2.76(4)
C11	.H4	2.50(4)	H4	.C11	2.50(4)
H7	.N1	2.8000	H9	.H10	2.3400
H7	.H8	2.3000	H10	.H9	2.3400
H8	.H9	2.3200	H10	.O4_g	2.8900
H8	.H7	2.3000	H10	.N3	2.8000
H8	.C12_f	3.0800	H12	.O2	2.7300
H8	.O3_f	2.6000	H12	.O4_e	2.8800
H9	.H8	2.3200	H12	.H4	2.0700

Table S9 - Hydrogen Bonds (Angstrom, Deg)

Crystal Data for TBZ- Formic Acid Solvate

N1	-- H1	.. O2	0.87(2)	1.76(2)	2.620(2)	171(2)	.
N3	-- H3	.. O1	0.82(2)	1.89(2)	2.684(2)	161(2)	4_455
O4	-- H4	.. O1	0.90(4)	1.69(4)	2.585(3)	178(4)	.
C1	-- H1A	.. O3	0.9300	2.5100	3.070(3)	119.00	3_666
C2	-- H2	.. O2	0.9300	2.5100	3.325(3)	147.00	.
C8	-- H8	.. O3	0.9300	2.6000	3.495(3)	162.00	2_745

Translation of Symmetry Code to Equiv.Pos

a =[1655.00] = [1_655] = 1+x,y,z

b =[3666.00] = [3_666] = 1-x,1-y,1-z

c =[4455.00] = [4_466] = -1+x,1/2-y,1/2+z

d =[1455.00] = [1_455] = -1+x,y,z

e =[1455.00] = [1_455] = -1+x,y,z

f =[2745.00] = [2_745] = 2-x,-1/2+y,1/2-z

h =[1655.00] = [1_655] = 1+x,y,z

i =[4654.00] = [4_665] = 1+x,1/2-y,-1/2+z

j =[1455.00] = [1_455] = -1+x,y,z

k =[2755.00] = [2_755] = 2-x,1/2+y,1/2-z

m =[1655.00] = [1_655] = 1+x,y,z
